# Optical characterization of the liver tissue affected by fibrolamellar hepatocellular carcinoma based on internal filters of laser-induced fluorescence

**DOI:** 10.1038/s41598-022-10146-7

**Published:** 2022-04-12

**Authors:** Marzieh Amani, Ali Bavali, Parviz Parvin

**Affiliations:** Department of Energy Engineering and Physics, Amirkabir University of Technology, 15875-4413 Tehran, Iran

**Keywords:** Biological fluorescence, Tissues, Fluorescence spectroscopy

## Abstract

Laser-induced fluorescence (LIF) spectroscopy has recently gained regards for diagnosis of the cancer in various tissues of the human body. This method in its conventional form, when used for assay of highly scattering media, encounters a lot of noise due to multiple scattering and inner filter effects which overshadows the sensitivity and specificity of the method. Here, angular dependence of the LIF spectral shift due to the reabsorption events have been investigated for characterization of the bio-tissues. The aim was to determine the tissue morphological changeovers due to the cancer progression. The assessment of a rare type of the liver cancer i. e. fibrolamellar hepatocellular carcinoma revealed the significant difference in optical anisotropy of the parenchyma and liver tumor. As a result, utilizing LIF spectroscopy as a fast, highly sensitive and easy-to-use method one can evaluate the optical anisotropy for diagnosing tissues during the cancer progression.

## Introduction

Light interaction with biological tissues has received growing attention due to its potential for non-destructive study and treatment of diseases^1–4^. Though the optical interactions are widely investigated in plants^5,6^, animals^7,8^, and human tissues^9–11^, in most of the experimental reports biological tissues have been considered optically isotropic^10,12–14^. However, few studies have been performed on the anisotropic optical responses of the bio-tissues to acknowledge that it can be a proper optical parameter in diagnosing tissue lesions^7,15–19^. In our recent study, enhanced backscattering (EBS) technique was utilized to discriminate healthy from the cancerous tissues based on their distinct anisotropic optical responses^20^. Though the potency of EBS technique as a fast and non-destructive tool for ex-vivo determination of the tissue optical anisotropy was affirmed, it is required to search for a simple method due to the high complexity and sensitivity of the EBS setup to environmental conditions.

Laser-induced fluorescence (LIF) is an offered optical spectroscopic technique with high accuracy and simplicity^21–25^, that has been used in various fields such as biochemistry^26^, biophysics^27^, medicine^28,29^, genetic analysis^25^, and biotechnology^30^. LIF spectroscopy as a quick technique with less destructive effects has been employed in order to diagnose cancer^31,32^. In this method, the incident laser photons are absorbed by the fluorophores and then fluorescence emission occurs at higher wavelengths due to the Stokes shift^23,25^. It is noticeable that in scattering media such as biological tissues, various effects such as dynamic quenching, resonance energy transfer, and multiple scattering events strongly affect the intensity and spectral properties of the emitted fluorescence^32–34^. For this reason, LIF technique in its conventional form i.e. the measurement and analysis of the fluorescence intensity, could not provide reliable information for diagnosing the scattering media.

Fibrolamellar hepatocellular carcinoma (FLHCC) is a rare type of primary hepatocellular carcinoma (HCC) without specified symptoms, which mainly affects young people with an average age of 25 years^35^. Since FLHCC has genetic etiology and most of the infected people did not have any chronic liver disease, it can only be diagnosed in advanced stages^36^. Moreover, due to the rarity of FLHCC, there is no chemotherapy standard for therapy, and the liver resection is the most optimal way to treat disease despite its high recurrence rate^35^. Utilizing a prism-coupling technique and total internal reflection model, Giannios et al. investigated the wavelength dependence of the refractive index as a marker of discrimination between normal liver tissues and tumors^16^. They examined 450, 532, 632.8, 964 and 1551 nm wavelengths and revealed the higher refractive index of the liver’s normal tissue with respect to the tumors. Carneiro et al. also studied the wavelength dependence of the normal and cancerous liver tissues using prism-coupling technique besides direct measurement of the scattered and transmitted laser beam utilizing integrating sphere and spectrometer. For both tissues, they have reported the decrease in refractive index, absorption and scattering coefficients, and increase in anisotropy with the wavelength^17^. Germer et al. have measured the scattering coefficients as well as the anisotropy factors of human liver tissue and colorectal liver metastases at 850, 980, and 1064 nm wavelengths to show that the liver tumor have a lower anisotropy factor and scattering coefficient than the healthy liver tissue^37^. They utilized a grid monochromator in Czerny-Turner configuration with a stepper motor to select desired wavelength from the emission of a Mercury arc lamp. A double integrating sphere with silicon photodiodes and an integrated preamplifier system supported by a lock-in amplifier technique was used to collect and detect the scattered radiation field^37^. Though the reports suggest optical anisotropy as a reliable parameter for diagnosing tissue structural deformations, utilized methods are not widely practical due to their complexity and sensitivity to intensity fluctuations induced by environmental conditions. Therefore, search for a simple handheld and fast optical characterization tool would be of particular importance in order to timely diagnosis of aggressive cancers.

In present report, the optical anisotropy of the healthy and cancerous (FLHCC type) tissues of the liver organ is investigated based on the angular dependence of the secondary inner filter effect (2^nd^-IFE) of LIF. Here, LIF spectral shift is considered as main optical parameter because unlike the fluorescence intensity, it is less dependent on ambient noises. The purpose was to develop a fast, non-destructive and easy accessible tool for real-time tissue diagnosis during the cancer progression.

## Optical model

### Fluorescence reabsorption as IFE

In literature, reabsorption of the fluorescence emission by the ambient absorbers is known as secondary inner filter effect (2nd-IFE)^25,38^. According to Beer-lambert's law (Eq. 1), the rate of reabsorption events and the corresponding reduction of the fluorescence intensity, F_0_(λ), after travelling the optical path-length, ${l}_{t}$, in a medium containing fluorophores, depends on the density of the non-excited (ground state) fluorophores $N_{dye}^{G} \left( l \right)$ and the reabsorption cross-section σ_reabs_.(λ). The latter is associated with the fluorescence Stokes shift which determines overlapping area between the absorption and fluorescence spectra^20^.

1 $$ F\left( {\lambda ,l_{t} } \right) = F_{0} \exp \left[ { - \int_{0}^{{l_{t} }} {\sigma_{reabs.} } \left( \lambda \right)N_{dye}^{G} \left( {l^{{\prime }} } \right)dl^{{\prime }} } \right]. $$

On the other hand, based on diffusion model of the light propagation in scattering media (such as epithelial tissues), photon travelling path length *l*_*t*_ mainly depends on both density and associated scattering cross section of the scattering sites^20^. Pursuant to Eq. (2), *l*_*t*_ is determined by two factors: scattering cross-section of each scattering site, σscat.(λ;x,y,z) and the number density of the scatterers in medium, Nscat. (x, y, z):

2 $$ l_{t} \propto \sigma_{scat.} \left( {\lambda ;x,y,z} \right)N_{scat.} \left( {x,y,z} \right). $$

According to Eqs. (1) and (2), the higher the travelling length and/or density of the non-excited molecules and/or the reabsorption cross-section, the more reabsorption events (as 2nd-IFE) and subsequent reduction of the detected fluorescence intensity^20^.

**Figure 1:**
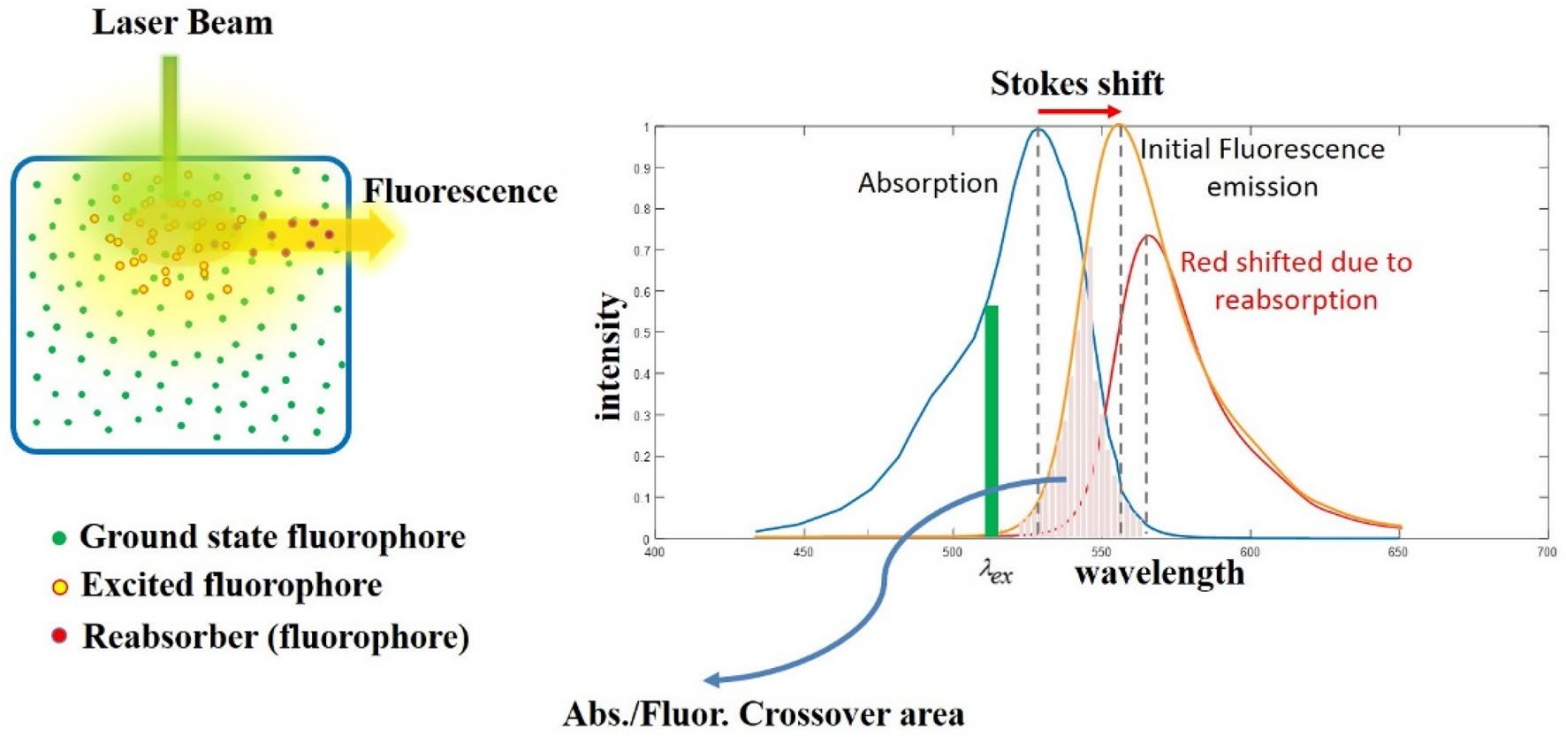
Left: Typical fluorescent medium excited by a narrow laser beam and associated fluorescence emission that is detected at right angle setup (90°); Detected LIF emission is subjected to reabsorption events. Right: Typical absorption–emission spectra of Rd6G dye solution and associated crossover area (hatched). Detected fluorescence peak wavelength is red shifted respect to the initial one, because the reabsorption events preferentially occur at shorter wavelengths of the fluorescence spectrum.

In the case of fluorophores with significant overlap between the absorption and emission spectra (such as Rhodamine 6G (Rd6G)^21^), since the reabsorption events preferentially occur at shorter wavelengths of the fluorescence spectrum, the reduction in intensity is stronger for those wavelengths in the crossover region as demonstrated in Fig. 1. As a result, another consequence of the reabsorption events is the non-intrinsic spectral red shift of the fluorescence peak, which has already been examined in detail^21,34^.

### Angular dependent IFE

Figure 1 (left) demonstrates a typical model for a medium with homogeneous distribution of fluorophores. Due to the local pumping of medium with a narrow laser beam, incident photons form an excitation volume with non-homogeneous distribution of the excited molecules in front of the medium.

As the density of the incident photons decreases with distance from the original laser beam pathway, population of the excited molecules would be reduced by increasing the detection angle (respect to the laser beam direction). Therefore, the probability of reabsorption by non-excited molecules within the FOV increases at larger detection angles which leads to the angular dependence of the fluorescence IFE. As a result, according to Eq. (1), the emission intensity decreases by increasing the angle of detection. It should be emphasized that similar to the optical model represented in Fig. 1, the tissue samples under examination did not have radial symmetry (around an axis perpendicular to the laser beam direction), which could be considered as an inevitable factor for the decrease in intensity with increasing angle. However, since the geometry of the measurement setup was the same for both healthy and cancerous tissues, this factor is not intended to compare the results for two cases of healthy and cancerous.

It is worth noting that the greater the scattering strength of the medium, the larger the excitation volume^33,34^. Previous reports on the scattering of light in colloidal suspension of nanoparticles in dye solutions show that as the scattering strength of the medium increases and the photons diffuse over a larger volume of the medium, the number of the non-excited molecules decreases that leads to reducing the reabsorption events and alleviation of the corresponding angular dependence of the detected intensity.

### Optical anisotropy

**Figure 2:**
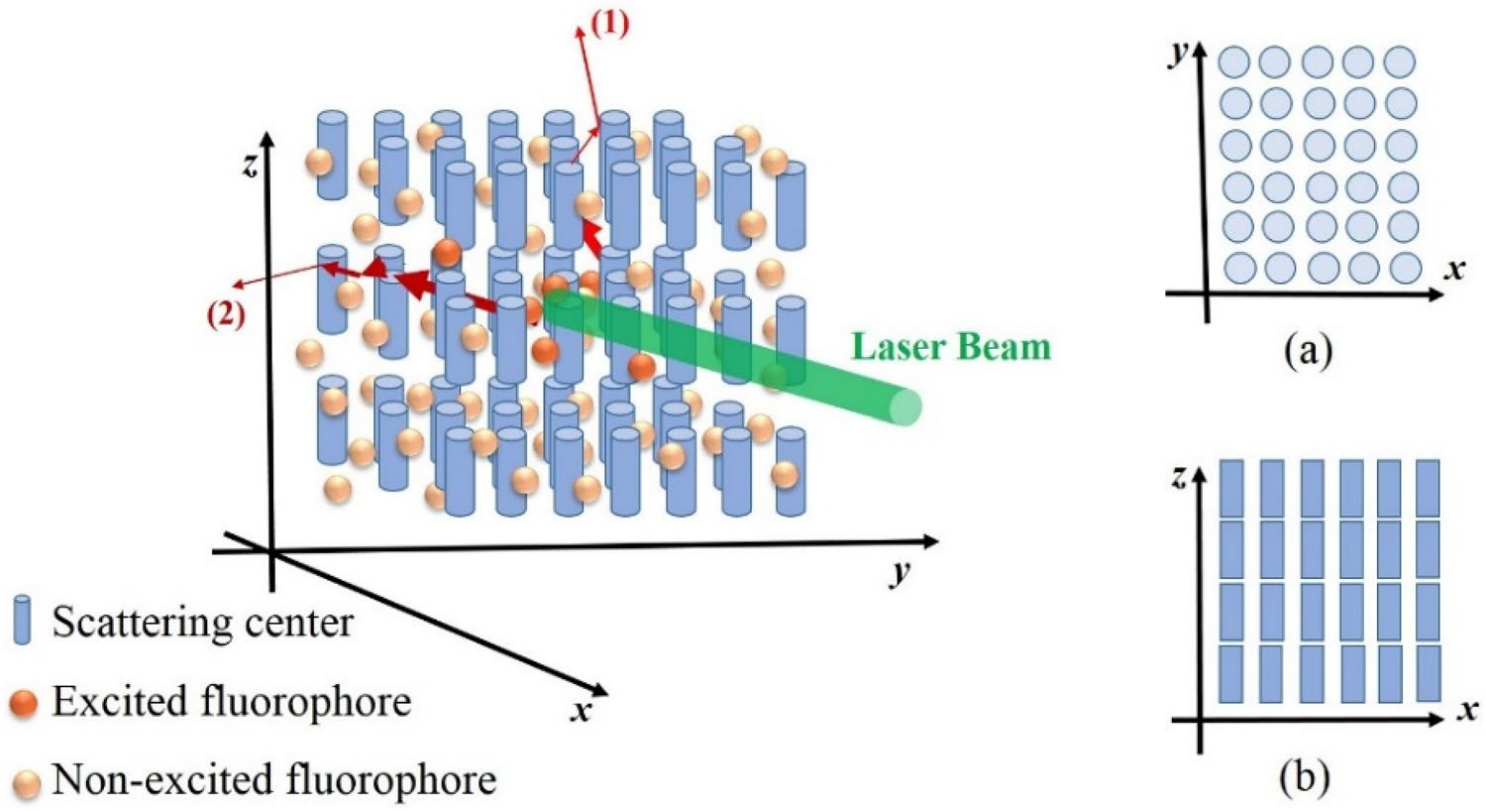
Typical model of the optically anisotropic medium that contains cylindrical-shape particles orderly dispersed in a homogeneous dye solution. The medium is locally pumped by a narrow laser beam along x-direction. Two pathways (1) and (2) show typical fluorescence photons emitted in the z and y directions, respectively. Inset (**a**) and (**b**) indicate the cross sections of the medium that photons (1) and (2) encounter respectively.

In the previous section, angular profile of the LIF intensity was introduced as a result of the 2^nd^-IFE, and its dependence on the scattering strength of the medium was explained. In an optically isotropic medium, this profile should not change significantly by rotating the medium around the axis passing through the excitation laser beam direction. Because in such a medium, the mean scattering length (or corresponding diffusion coefficient) is the same for light propagation in all directions. Although many optical media such as solutions and colloids of spherical nanoparticles in dye solutions are optically isotropic, several biological tissues are not necessarily isotropic^18,19^. Figure 2 illustrates typical model of the anisotropic optical medium. It consists of cylindrical-shape particles that are orderly dispersed in a homogeneous dye solution. The laser beam enters the medium in x direction, excites molecules in its path, and fluorescence emission is emitted in all directions. Also, the two pathways (1) and (2) show typical fluorescence photons emitted in the z and y directions, respectively. As explained in the previous section, if the fiber optics probe detects fluorescence emission at different angles on the x–y plane, an uneven intensity profile is recorded. The point is, if the optical fiber scans the angles on the x–z plane, the intensity profile it records is noticeably different. The reason for this can be explained based on comparison of the two cross-sections of the medium shown in inset of Fig. 2.

Figure 2-insets (a) and (b) indicate the cross sections of the medium that photons (1) and (2) encounter respectively. As can be seen, if we consider the scattering cross section of each particle as a function of its size and shape, the scattering strength of the medium for pathway (1) is completely different from that of pathway (2).

Since the scattering cross section of each particle is a function of its size and shape, this difference indicates the variation in the scattering strength of the medium for emitted light in two directions (1) and (2). In other word, the medium which contains orderly oriented scattering particles (with anisotropic shape) is expected to be optically anisotropic. As a result, IFE (θ) which determines the angular profile of the detected LIF intensity would be dependent on the spatial orientation of the medium respect to the x–y plane.

## Methods and experiments

### Tissue sample preparation

Healthy parenchyma and cancerous liver tissues of 5 patients were studied. Liver lesion was associated with fibrolamellar hepatocellular carcinoma (FLHCC) and was assigned as carcinoma in situ after biopsy. Tissues were prepared from Imam Khomeini Hospital, and were kept in 10% neutral buffered formalin immediately after biopsy to prevent deformations. All patients provided informed consent to participate in the study. Experiments on tissues accomplished ex-vivo in accordance with relevant guidelines and regulations. The protocol for the use of tissues was consented and approved by the ethics committee of Imam Khomeini medical Centre. For each patient, 8 samples containing 4 adjacent pieces of normal and 4 adjacent pieces of cancerous lesions were investigated. The parenchyma pieces were picked up from the areas far enough from the lesion margin to avoid the conterminous lesions. Coborder pieces were examined without changing their initial position respect to each other. The tissue slices were cut to dimensions of 20 × 20 × 5 mm (approximately). The dye used was Rd6G (C_27_H_29_ClN_2_O_3_) with typical concentration of 0.1 M. Ethanol was used as the solvent of Rd6G. Each tissue was stained in Rd6G solution for 30 min.

### Laser induced fluorescence spectroscopy

**Figure 3:**
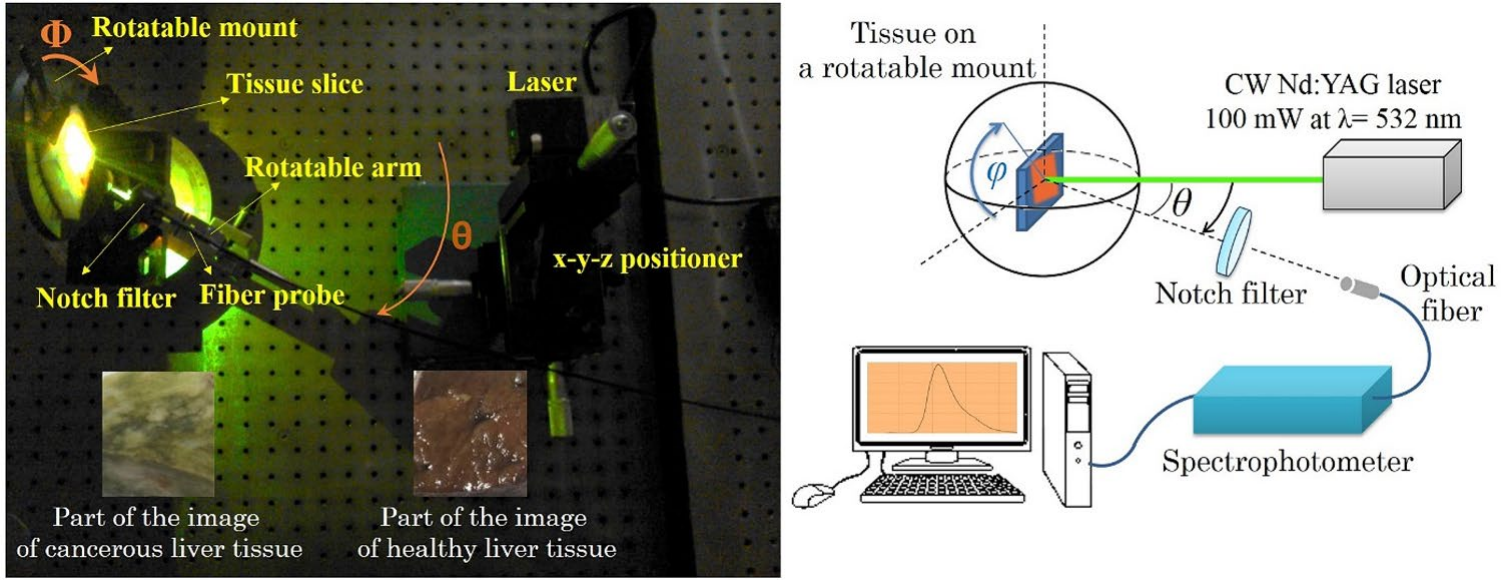
Angular measurement setup.

The second harmonic generation (SHG) of 100 mW CW-Nd:YAG laser with beam diameter of 0.5 mm at 532 nm was used to illuminate the tissue samples. The neutral density (ND) filter was employed to control the laser beam intensity for optimum illumination power. A notch filter (Edmond Optics) with FWHM = 26.6 nm and transmission range of 400–700 nm was utilized to remove the scattering of incident laser photons from the tissues. The LIF emission was collected using a UV–visible-NIR spectrophotometer, Avantes AvaSpec2048, with a spectral resolution of 0.4 nm (within the spectral range of 200–1100 nm) and SMA-905 UV600/660 fiber probe with NA = 0.22 (acceptance angle of 12.7°). Figure 3 illustrates the the measurement setup. Tissue slices were placed in the center of a rotatable mount. The fiber probe was placed at a fixed distance of 5 cm from the center of the samples mounted on a rotatable arm with a rotation accuracy of 0.5° along the radial axis (θ). The rotating mount was oriented in four orientations: φ = 0°, 45°, 90°, and 135° respect to the horizontal axis of the rotatable mount (φ = 0^◦^ was allocated to the 1st position of the given tissue on rotatable mount). The spectra were recorded at θ = 10°–90° with 10-degree steps for each orientation (φ). In general, local pumping with 0.5 mm laser beam spot provides non-homogeneous population of the excited molecules that leads to angular dependence re-absorption rate (as IFE (θ)) and the corresponding angular (θ) dependence spectral red shift^34^. Such an angular pattern was measured for different orientations of the sample (φ) to assess the optical anisotropy of the tissue under investigation.

**Figure 4:**
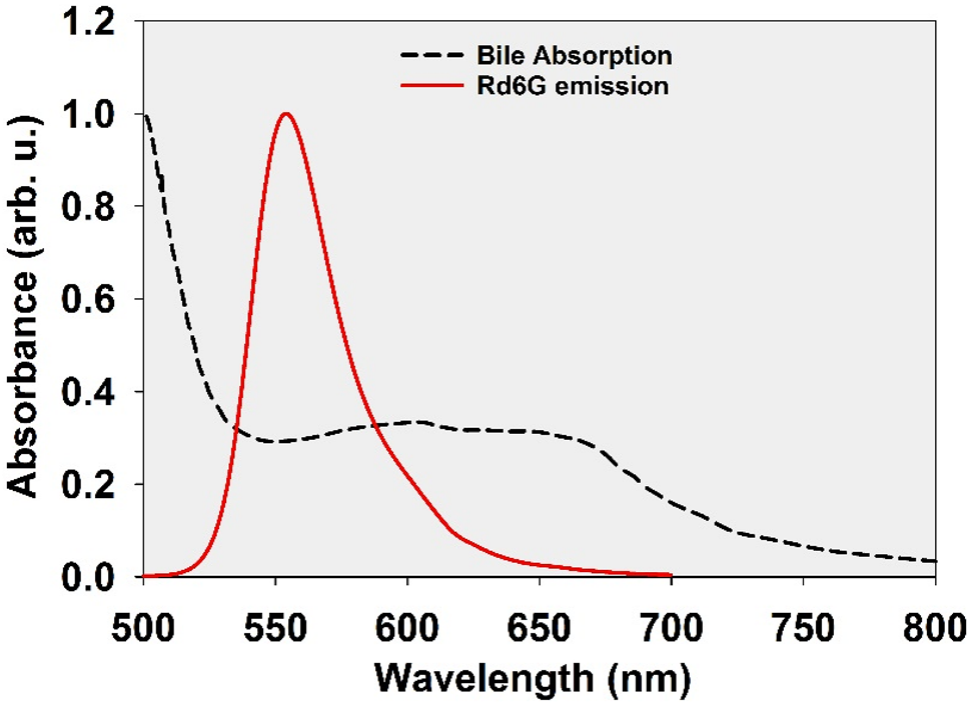
Absorption spectrum of Bile and fluorescence spectrum of Rd6G (Both normalized).

It is worth noting that the occurrence of significant reabsorption requires two preliminary conditions: the first is the small Stokes shift of the fluorescence spectrum and the second is the local pumping of the medium by a narrow beam of photons. The first condition is necessary for the self-absorption event, and the second condition must be met so that enough of the ground state absorbers are in the path of the fluorescence photons produced within the tissue to be able to reabsorb. The first condition is well established for Rd6G with typical Stokes of ~ 25 nm, and the use of the LIF technique is to satisfy the second criterion. On the other hand, none of the endogenous fluorophores in the human tissues have such a small Stokes shift^38^. Therefore, the results obtained in this study cannot be achieved by Laser induced auto-fluorescence of the tissues. In addition, though the bile of a healthy liver can absorb within the visible spectral range, it cannot cause LIF emission to be red shifted. Looking at the absorption spectrum of Bile^39^ in the fluorescence spectral range of Rd6G (Fig. 4), it can be concluded that this absorber, despite reabsorption of Rd6G fluorescence, cannot cause spectral redshift due to uniform absorbance within the spectral overlapping region (almost equally at all wavelengths of the Rd6G LIF spectrum) and only reduces the fluorescence intensity.

### Spectra analysis

Statistical analysis of the spectra was accomplished by standard paired t-test based on Shapiro–Wilk normality test in SigmaPlot v.14 package. Moreover, in order to evaluate the anisotropy parameter (which is defined based on the anisotropy in reabsorption events) as an accurate classifier, receiver operating characteristic (ROC) analysis has been used.

## Results

**Figure 5:**
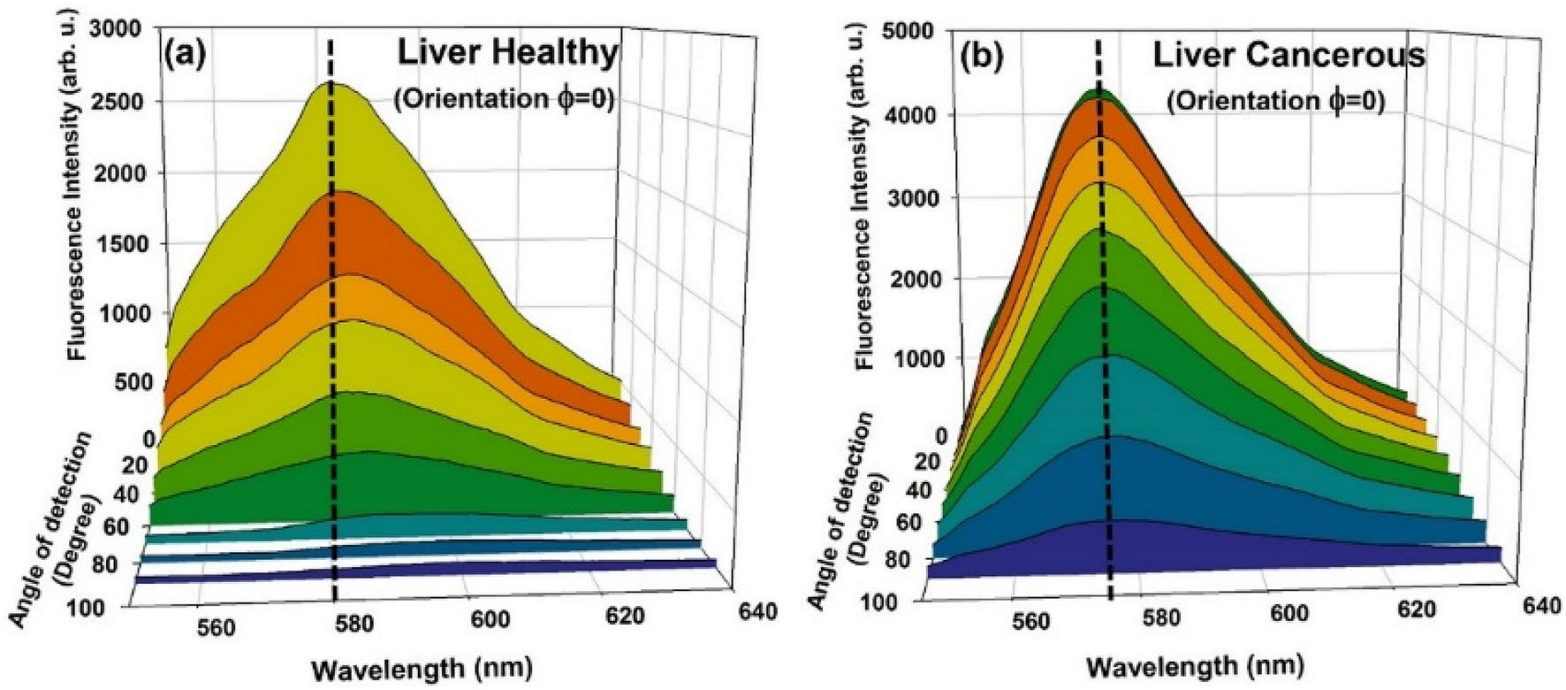
LIF spectra detected at θ = 10°–90° due to excitation of stained (**a**) healthy and (**b**) cancerous liver tissues. Tissue is oriented at $\mathrm{\varphi }$ = 0°.

Typical LIF spectra of healthy and cancerous tissues detected at various angles θ = 10°–90° with 10-degree steps, for tissue orientation φ = 0° is shown in Fig. 5(a) and (b). A comparison of the spectra indicates that: (1) at each detection angle (θ) LIF intensity for healthy tissue is significantly less than that of cancerous, (2) the rate of reduction of the intensity with elevating θ is greater for healthy tissue, (3) for healthy tissue, spectra are spectrally red shifted by increasing the angle of detection.

Similar trends (albeit with differences in detail) were observed for the measured spectra of both healthy and cancerous tissues in other orientations (φ = 45°, 90°, and 135°). In order to investigate these results more attentively, peak intensity and the wavelength LIF peak (λ_max_) were determined and plotted versus the detection angle (θ).

**Figure 6:**
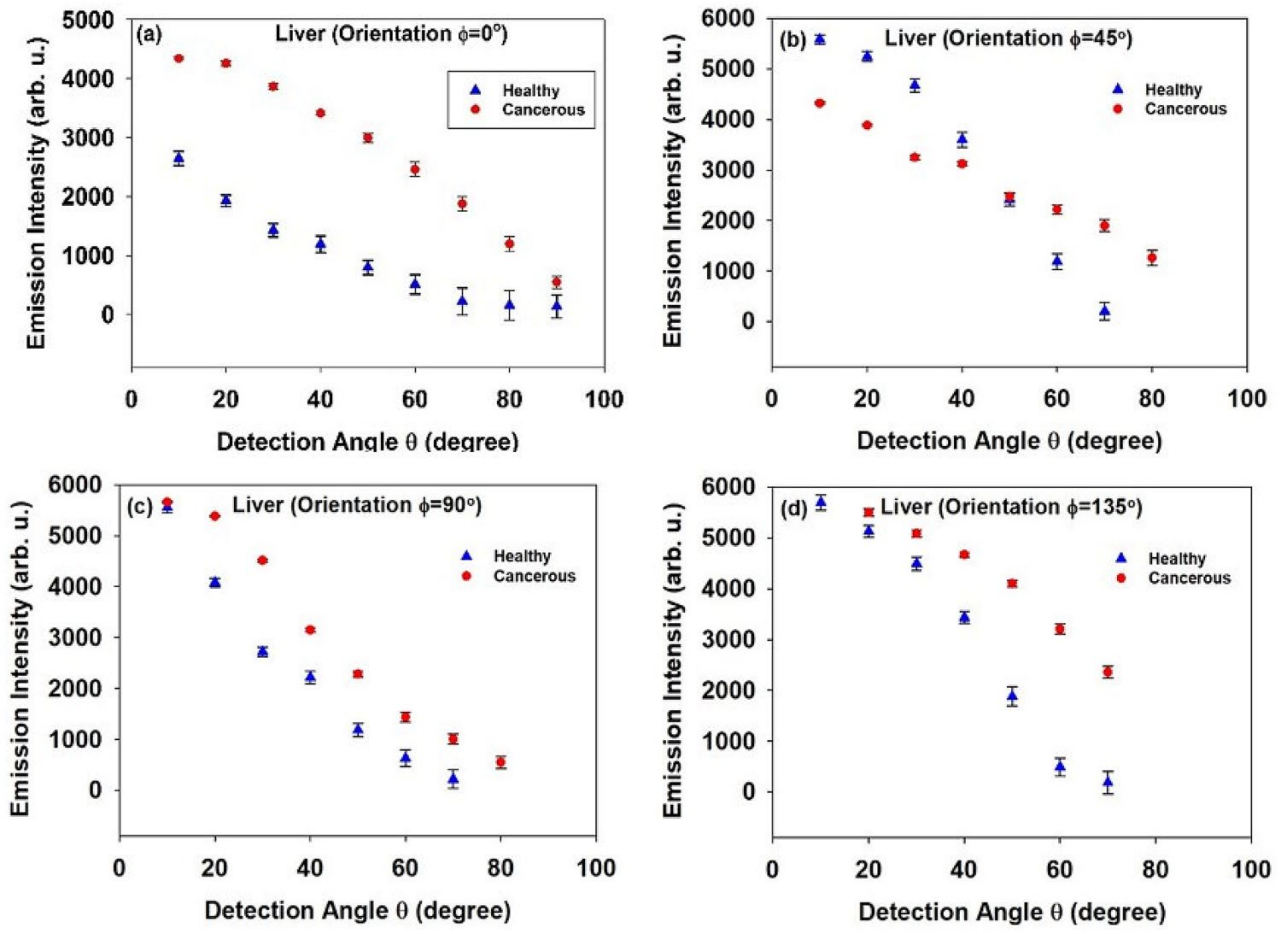
Intensity of the LIF emission in terms of detection angle for two healthy and cancerous Liver tissues at different orientations: (**a**) φ = 0°, (**b**) φ = 45°, (**c**) φ = 90°, and (**d**) φ = 135°.

Figure 6(a)–(d) depict intensity of the LIF spectra versus detection angle for healthy and cancerous liver tissues. For both tissues, at all orientations (φ) the intensity decreases with increasing detection angle (θ).

**Figure 7:**
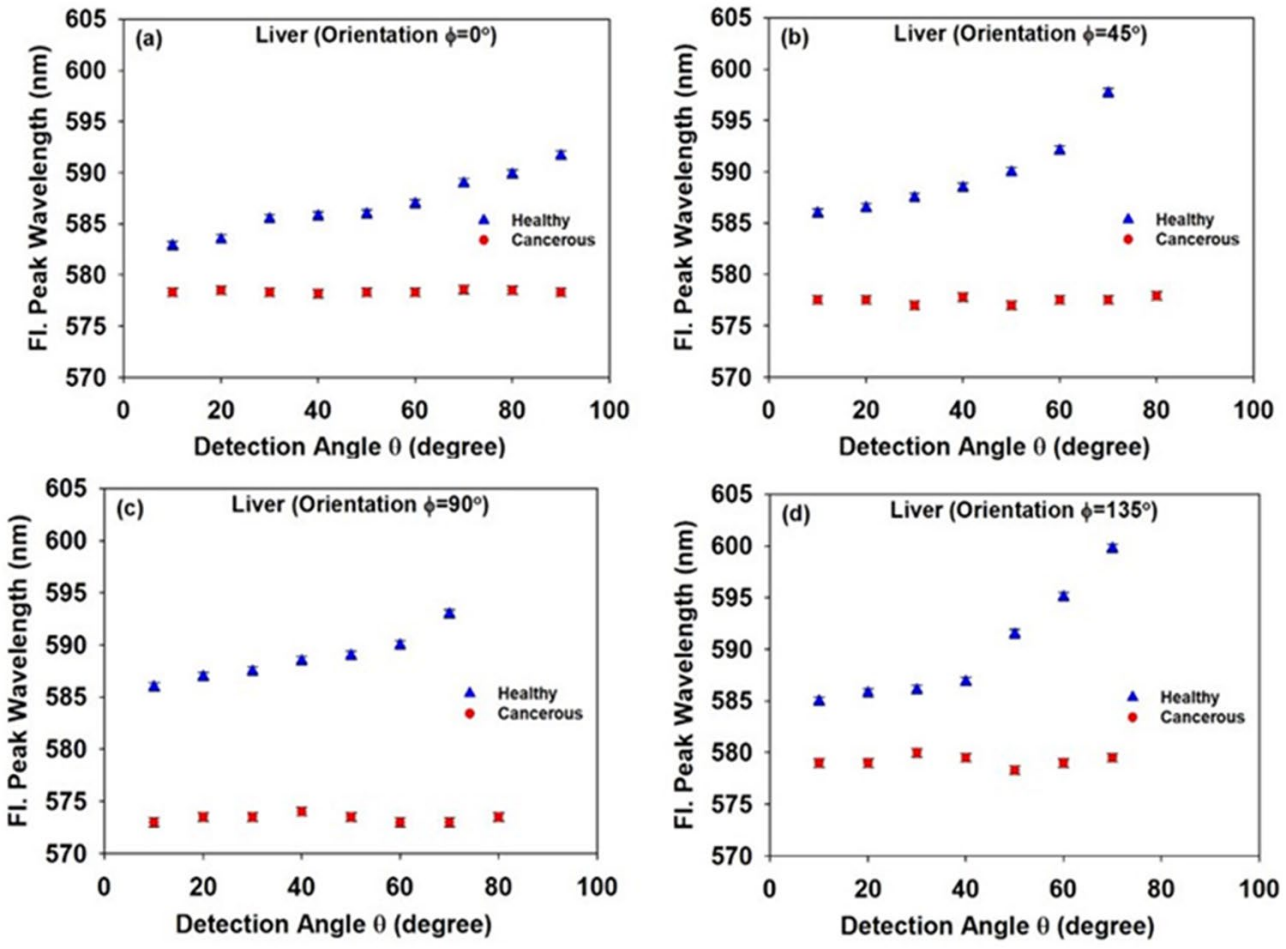
Wavelength of the LIF peak (λ_max_) in terms of detection angle ($\theta$) for healthy and cancerous liver tissues at different orientations: (a) φ = 0°, (b) φ = 45°, (c) φ = 90°, and (d) φ = 135°.

Figure 7(a)–(d) illustrate wavelength of the LIF peak (λ_max_) against the angle of detection (θ) for both healthy and cancerous liver tissues at different orientations (φ). It is clearly shown that the LIF peak wavelength is shorter and more isotropic in the case of cancerous tissue. In the case of healthy tissue, λ_max_ increases at larger detection angles for all four orientations. However, it is angularly preserved for all orientations of the cancerous tissue. It is worth noting that the reduction in emission intensity is due to the several factors e. g. reabsorption events, multiple scattering and elongation of the optical path length. However, the spectral shift depends only on the reabsorption rate that is identified as 2^nd^-IFE in the measurement setup. That is why the spectral shift is introduced here as a reliable parameter for discrimination of tumor from the healthy tissue.

As the scattering strength determines the reabsorption rate and the resulting spectral shift, total angular spectral shift (Δ_ss_ = Δλ_max_ (θ) = λ_max_ (90°)−λ_max_ (0°)) is introduced as an indicator of the tissue scattering strength, and being distinct in different orientations, Δ_ss_(φ), implies the optical anisotropy of the tissue. Maximum spectral shift value $\Delta {\lambda }_{max}(\theta )$ due to reabsorption of the fluorescence emission is presented in Table (1) for different tissue orientations. In addition, standard deviation (SD) of the measured Δλ_max_ (θ) in different tissue orientations is calculated to show how associated values have been spread out over the spatial directions. In this way, SD could be a proper indicator of the optical anisotropy of the given tissue.

According to data displayed in Table (1), for all orientations of the tumor, Δλ_max_(θ) is much less than that measured for healthy tissue, meaning that the IFE has occurred much less frequently in cancerous tissue. Note that the lower the spectral shift in the cancerous tissue is in agreement with relatively higher intensity of the corresponding LIF spectra compared to the healthy tissue (see Fig. 6). In addition, the amount of variation of the Δλ_max_(θ) associated with the different orientations is negligible which implies a decrease in tissue anisotropy due to the cancer progression.

The values of Δλ_max_(θ) (as the indicator of the scattering strength) and associated SD (as the indicator of the optical anisotropy) for all healthy and tumor samples were statistically analyzed using the standard paired t-test based on Shapiro–Wilk normality test in SigmaPlot v.14 package. The assay revealed that in general, Δλ_max_(θ) is larger (i. e. scattering strength is lower) at the 99.2% confidence level and SD is larger (optical anisotropy is higher) at the 97.5% confidence level in healthy liver tissue than the cancerous one. The results can be attributed to the morphological changes of the liver tissue during the cancer progression based on which, the increase in scattering strength and the decrease in optical anisotropy are explained separately in the following sections.

### Scattering strength

During the cancer progression, cell nuclei become enlarged and proliferation of the cells happens which causes the number of scattering centers to be increased. Moreover, extracellular structure becomes dense with lots of fibers and collagens thus, the scattering strength is expected to be enhanced as reported previously^20,36,37,40^. Consequently, laser photons entering the tissue will experience more scattering events leading to an enlarged excitation volume and consequent increase in the excited population. Due to this event, reabsorption in the cancerous tissue decreases because the reabsorption can only take place outside the excitation volume by ground state Rd6G molecules. Therefore at all detection angles (θ), LIF spectrum of the cancerous tissue has shorter peak wavelength respect to the healthy one.

About 70–85% of the liver volume is occupied by parenchymal hepatocytes^41^. However, in the case of FLHCC, liver biopsy shows heterogeneous sheets, nests, and trabeculae of tumor cells separated by dense collagen bundles. The large polygonal tumor cells have coarsely granular cytoplasm with large vesicular nuclei^35^. These heterogeneities lead to enhance the refractive index contrast in this tissue, which results in increasing the number of scattering events and consequently a significant increase in scattering strength.

### Optical anisotropy

Typical healthy epithelial tissue mainly consists of orderly oriented basic cells in the form of regular array^20^. Therefore, based on the explanations provided in Sect. 2.C., photons propagating within the healthy tissue face different scattering strength along orthogonal directions due to the difference in the cross sectional area^18–20^. Therefore, the travelling length *l*_*t*_ and the associated reabsorption rate is expected to be dependent on the direction of the photon propagation. As a result, in our measurement setup, the angular distribution of the fluorescence peak wavelength λ_max_(θ) would be different at disparate tissue orientations (φ). The above argument does not apply for cancerous tissues. Ordered array of basic cells in healthy tissue becomes disordered due to the cancer progression to form an optically isotropic medium created by non-aligned cells that are randomly distributed along different spatial directions. This is in agreement with the report of Germer et al. that the optical anisotropy is reduced in the cancerous liver tissue^37^. Accordingly, the amount of fluorescence spectral shift (due to the IFE) would be angularly preserved as revealed in Fig. 7. It was also elucidated from the Table (1) that at all orientations (φ) the LIF peak wavelength is higher in healthy tissue than that of tumor.

### Anisotropy as a classifier

**Figure 8:**
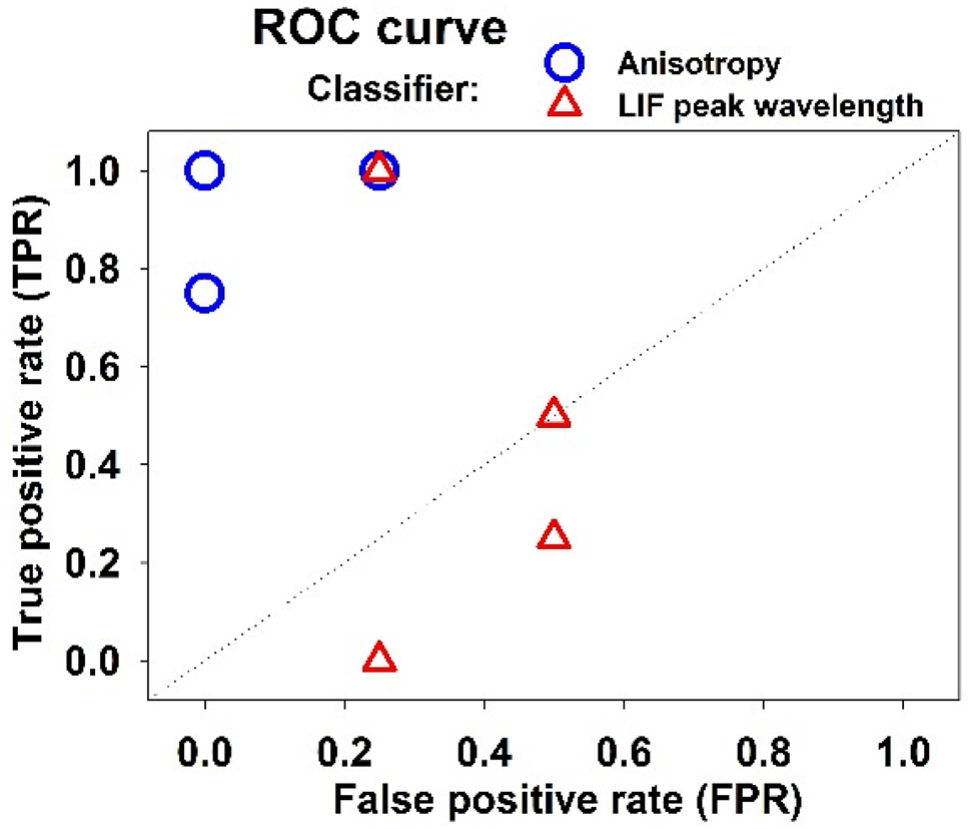
ROC in order to evaluate the anisotropy parameter as an accurate classifier.

Although the number of FLHCC tissues was not enough to perform a statistical analysis, we established ROC curve for the data by considering the two LIF-based parameters: (i) LIF peak wavelength and (ii) optical anisotropy (i. e. anisotropy of spectral shifts). Assuming that the classifier is the anisotropy factor, for each of the 5 patients, if the SD related to each of the healthy and tumor pieces was more than 2, it was considered cancerous, and if it was less than 1, it was considered healthy (thresholds were selected according to Table 1). In the case of the fluorescence peak wavelength to be classifier, peak wavelength of the LIF emission detected at θ = 30° for a random tissue orientation was recorded for each patient. According to Fig. 7, if the peak wavelength was greater than 580 nm, the tissue was healthy and if it was less, the tissue was considered to be cancerous. As depicted in Fig. 8, the anisotropy parameter is more reliable for discriminating healthy and FLHCC tumors than the LIF peak wavelength.

**Table 1:** Total spectral shift (Δλ_max_(θ)) due to reabsorption of the LIF in liver tissue for different orientations.

	Δλ_max_(θ), nm				Standard deviation (SD)
Tissue-Type	φ = 0^°^	φ = 45°	φ = 90°	φ = 135°	
Liver-Healthy	8.8 ± 0.7	11.7 ± 0.5	7.0 ± 0.4	14.8 ± 1.3	3.41
Liver-Cancerous	0.4 ± 0.4	0.9 ± 0.4	1.0 ± 0.4	1.7 ± 0.4	0.53

## Discussion

The aim of this paper was to assess the potency of secondary inner filter effect (i.e. reabsorption events) regarding the LIF spectroscopy as a reliable criterion for ex-vivo assay of the tissue structural changes due to the cancer progression. Liver tissue affected by FLHCC is examined as a tumor. It is demonstrated that the liver tumor with fibrous stem cells is subject to a decrease in anisotropy and increase in scattering strength, in conformity with previous reports based on intricate optical measurements such as EBS technique or reflectometry. In general, results affirmed that the non-intrinsic LIF spectral shift due to the re-absorption events (as LIF’s 2nd-IFE) can be a credible factor for identification of the tissue morphological changeovers. Proofs conducted that the spectral shift parameter is mainly affected by the reabsorption events while the conventional LIF (which is based on the intensity analysis) is influenced by various environmental factors that can lead to significant errors. This makes LIF spectral shift analysis a superior technique to investigate the tissue optical anisotropy as a criterion in diagnosing the cancer progression. The findings would be of significance in development of novel optical methods for accurate biopsy, which is the most common method of diagnosis in this type of cancer. The method determines an optical feature of the tissue (optical anisotropy) that provides information somewhat different from that specified in biopsy (microscopic images of the tissue) and thus can be raised as a complementary technique to develop the tissue diagnostic techniques.

## Limitations

The present research was intended to provide novel perspective regarding the optical characterization of the bio-tissues (e. g. liver healthy and tumor tissues) utilizing LIF spectroscopy. The main limitation of the proposed technique is that it is limited to the in-vitro study of tissues soaked in exogenous fluorophores with small Stokes shift. For in-vivo examination, bio-compatible fluorophores with small Stokes shift e. g. Methylene blue or Indocyanine green should be used and a fiber laser is required for illumination of the desired tissues. This possibility would be a valuable topic for future researches. Another limitation arises from relatively small number of samples. FLHCC is a rare type of the Liver carcinoma and sufficient samples may not be found to achieve an accurate quantitative characterization. In fact, the main purpose of this study was to show the effect of the fluorescence internal filters on laser induced fluorescence spectroscopy of the bio-tissues and to introduce it as a criterion for tissue characterization (optical anisotropy). Thus, the results, rather than demonstrating the relatively accurate values of the optical characteristics in tissues associated with FLHCC, qualitatively demonstrate the potency of the proposed method to differentiate FLHCC from healthy tissue.

Certainly, by comparing more FLHCC samples with healthy ones as well as conventional HCC type and/or early cirrhosis liver tissues, it is possible to provide quantitative results to assess the accuracy and resolution of this technique, which could be a proper topic for future research.

## Data Availability

The datasets obtained by experiments and/or analyzed during the current research are not publicly available because we do not have consent from all patients to publish the raw data.
